# On the Mathematical Modeling of Slender Biomedical Continuum Robots

**DOI:** 10.3389/frobt.2021.732643

**Published:** 2021-10-05

**Authors:** Hunter B. Gilbert

**Affiliations:** Department of Mechanical and Industrial Engineering, Louisiana State University, Baton Rouge, LA, United States

**Keywords:** continuum robots, soft robots, dynamics, statics, mechanics, medical robotics

## Abstract

The passive, mechanical adaptation of slender, deformable robots to their environment, whether the robot be made of hard materials or soft ones, makes them desirable as tools for medical procedures. Their reduced physical compliance can provide a form of embodied intelligence that allows the natural dynamics of interaction between the robot and its environment to guide the evolution of the combined robot-environment system. To design these systems, the problems of analysis, design optimization, control, and motion planning remain of great importance because, in general, the advantages afforded by increased mechanical compliance must be balanced against penalties such as slower dynamics, increased difficulty in the design of control systems, and greater kinematic uncertainty. The models that form the basis of these problems should be reasonably accurate yet not prohibitively expensive to formulate and solve. In this article, the state-of-the-art modeling techniques for continuum robots are reviewed and cast in a common language. Classical theories of mechanics are used to outline formal guidelines for the selection of appropriate degrees of freedom in models of continuum robots, both in terms of number and of quality, for geometrically nonlinear models built from the general family of one-dimensional rod models of continuum mechanics. Consideration is also given to the variety of actuators found in existing designs, the types of interaction that occur between continuum robots and their biomedical environments, the imposition of constraints on degrees of freedom, and to the numerical solution of the family of models under study. Finally, some open problems of modeling are discussed and future challenges are identified.

## Introduction

**FIGURE 1:**
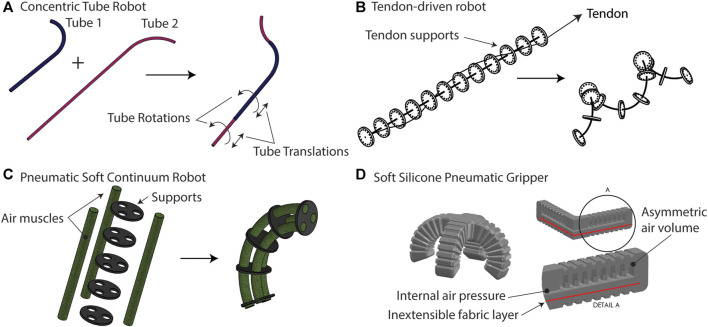
**(A)** Concentric tube robots are comprised of hard (metallic) tubes which are precurved and nested inside one another. Rotating and translating the tubes results in motion. **(B)** Tendon-driven robots use one or more tendons or cables to provide internal actuation forces that bend a flexible, slender rod. **(C)** Pneumatic soft continuum robots use soft air muscles, which extend or contract with internal air pressure, to create bending in a composite structure. The supports could be hard or soft materials. **(D)** A fully soft pneumatic gripper uses asymmetry introduced by an inextensible fabric layer and an asymmetric air volume to create four slender fingers which bend to wrap around objects.

Continuum robots use material deformation to move instead of joints. They may offer a technological solution to some of the difficult challenges of locomotion, perception, and manipulation found in a variety of unstructured and uncertain environments (Robinson and Davies, 1999). Biomedical applications have been a great motivator in the development of a wide variety of continuum and soft robots, ranging from surgery to therapy and other applications involving physical human-robot interaction. The great recent interest in these design paradigms stems from the observation that success in whatever form it is needed may be achieved without having complete control over the motion of a robot or its forces of interaction with the environment. In some cases, this is advantageous simply for reducing the complexity of engineered systems, and in other cases, performance may be increased beyond what is possible with rigid machines. Several excellent examples of this general principle come from tools of modern medicine. A flexible endoscope can navigate the intestines without a great degree of control over its own shape. The same is true for an intravascular catheter. In these examples, it is the particular combination of geometry and just the right amount of mechanical “softness” that facilitates the completion of the task. Beyond this snake-in-a-pipe approach to navigation, recent research has argued that physical compliance is advantageous in grasping, underwater swimming, robustness to collision, and locomotion on soft terrains where low ground pressure is required. The interested reader is referred to several review articles for a survey of the benefits, applications, challenges, and history of soft and continuum robots (Kim et al., 2013; Burgner-Kahrs et al., 2015; Walker et al., 2016; Cianchetti et al., 2018). Figure 1 shows four examples of continuum robot architectures which range from fully hard materials to fully soft and with composite structures in between these extremes.

Though there is not universal agreement on definitions, the term *continuum* robot is generally used to imply that motion is generated without identifiable kinematic pairs, while the term *soft* robot implies at least a greater degree of mechanical compliance, defined as the ratio of displacement to force, exhibited in response to environmental forces than traditional approaches to robotic interaction. Many soft robots are made of soft materials, which may be characterized in terms of a material parameter such as the modulus of elasticity (Majidi, 2014). Continuum robots made of harder materials can be designed to exhibit high or low mechanical stiffness to external forces depending on the design details.

Continuum robots are classified as under-actuated mechanisms (Spong, 1998). This statement is taken to mean that in a practical sense, and within the context of a pre-defined scope of possible robot-environment interactions, more information than can be collected by a finite set of actuator-collocated sensors is needed to describe the shape and motion of the robot to the degree of accuracy demanded by engineering specifications or by the roboticist’s preference. The practical sense of the definition is emphasized since even rigid robots with revolute or prismatic pairs must deform to a small degree when interacting with their environment via forces. All mechanical systems are underactuated when there exist flexible modes that are not actuated but which should be controlled (Spong and Laurent, 1997). It is well known that the analysis of dynamics of underactuated robots is significantly more complex than for regular, fully actuated robots (Jain and Rodriguez, 1993).

Beyond being under-actuated, the modeler of a continuum manipulator also frequently faces other challenges. Designs are often difficult to separate into “components” since the structure and the actuator may be the same physical body. Actuators based on pneumatics, hydraulics, and composite structures may not be as easily characterized as electric motors. Friction and hysteresis models may be needed to explain observed mechanics, and environments rich with expected contacts may require the solution of contact models based on theories of nonlinear complementarity. Additionally, the standard kinematic descriptions based on the rigid transformations in the special Euclidean group SE(3) are neither the most common approach to solid mechanics nor (necessarily) the most expedient approach to the description of solid continua undergoing deformation. With these considerations, one appreciates why the mathematical modeling of continuum and soft robots can be challenging.

This paper first reviews the state-of-the-art in the mathematical modeling of continuum manipulators having at least one “long” aspect in terms of its shape, which are termed *slender* in agreement with the mechanics literature. The goal of these models is to describe the dynamics (or statics) to relate actuator variables, other boundary conditions, and sensor measurements to the motion of the robot. The models are generally not concerned with other important aspects of robot design and analysis, such as repeatability, wear, safety, and other factors. For designs made of slender components, the motion of the robot is dominated by bending or beam-like deformations. This classification can be thought of as “arms,” “snakes,” or the individual “fingers” of a multi-fingered hand. Designs composed of individual components having this property are a natural extension, such as concentric tube robots (Mahoney et al., 2018) or multi-backbone continuum robots (Ding et al., 2013). For robots made of softer materials, such as the STIFF-FLOP designs, localized deformations may be complex, yet the dominant behavior is still beam-like (Cianchetti et al., 2014; Fraś et al., 2014). One of the goals of the work is to express the variety of methods encountered in the literature with a common notation. The review motivates a theoretical discussion rooted in the classical theories of solid mechanics. An analysis of the mechanics is used to support recommendations for future modeling efforts, with the conclusion that some choices for the model structure may result in better absolute model accuracy and efficiency (as quantified by the relationship between accuracy and dimensionality).

## Review of the State of the Art

Table 1 presents the unified nomenclature that will be used throughout this paper. In the discussion of other works, the original nomenclature has been changed to match what is shown. There are three primary considerations in any physics-based approach to modeling of solid continua: the adoption of kinematic hypotheses and coordinates describing the configuration of the body, the application of the laws of mechanics, and the selection of mathematical models that describe the behavior of materials (Sadati et al., 2019). Kinematic hypotheses alone allow the modeler to describe the geometry of the robot, but this alone is insufficient for most purposes because it does not reveal which configurations are possible or likely. The mechanics, which are formulated naturally as partial differential equations, provide the relationships between the kinematic degrees of freedom that indicate which path of configurations will be taken if particular conditions (actuation, environments, etc.) are imposed. Finally, the material models are needed to close the relationship between the kinematic degrees of freedom and the kinetic quantities related by the mechanics.

**TABLE 1:** Nomenclature used in this article.

Symbol	Meaning
p	Position vector of a point with respect to an inertial frame of reference ${ℱ}_{0}$
aℱ	Vector a resolved in Cartesian coordinates of frame ℱ. The basis is held fixed if a derivative is taken, i.e. if $a=x_{i}d_{i}$ and $d_{i}$ are the unit vectors of ℱ , then ∂saℱ=(∂sxi)di
${ℱ}_{i}$	Frame of reference i . ${ℱ}_{0}$ is an inertial frame
s	Arc length coordinate
t	Time coordinate (may be real time or an arbitrary parameter describing changes in configuration depending on context)
$d_{1},\;d_{2},\;d_{3}$	Director vectors of a framed curve
$g_{b},\;R_{b},\;p_{b}$	Transformation in SE(3) consisting of rotation operator $R_{b}\in SO\left(3\right)$ and displacement $p_{b}$ describing the transformation between ${ℱ}_{0}$ and ℱ(s) along a framed curve
$q_{i}$	A generalized coordinate for the $i^{th}$ degree of freedom
$\partial_{\alpha }\left(\cdot \right)$	Partial derivative operator with respect to variable α
u, v	Strain variables in the special Cosserat rod description
D	Subset of the real line, domain of the arc-length parameter for a rod
${\left(\cdot \right)}_{\times }$	Canonical mapping ${\mathbb{R}}^{3}\to so\left(3\right)\subset {\mathbb{R}}^{3\times 3}$ , $a_{\times }b=a\times b$
$u_{k}d_{k}$	Summation over repeated indices implied
$\tau_{j}$	Actuator value j
$x_{j}$	Task-space coordinate
$\dot{q}$	Time derivative of q

### Kinematic Descriptions

The forebears of continuum manipulators are the hyper-redundant robots, defined as those having a large (or infinite in the case of continuum robots) relative degree of redundancy (Chirikjian and Burdick, 1994). In any robot with material deformation which is substantial with regard to the kinematics or dynamics, both the relative degree of redundancy and the degree of under-actuation are theoretically infinite since the configuration space is infinite-dimensional. Here the usual definition of a robot configuration is used: “a complete specification of the location of every point on the robot” (Spong et al., 2006). There have been two primary methods to date of describing the configuration of continuum and soft robots: the curve-based description and the general continuum description.

#### The Curve-Based Description

**FIGURE 2:**
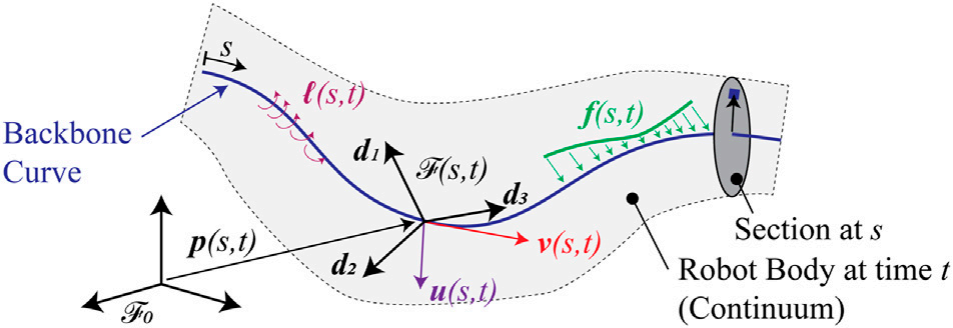
Mathematical setup of the curve-based kinematic description of slender continuum robots.

The state of the art curve-based description is that of the special Cosserat rod (Antman, 2005). Figure 2 depicts the curve, its relationship to a solid body, and the quantities that are associated with the curve and the boundary conditions of a mechanical model. The elongated form of many continuum manipulators leads naturally to the concept of the “backbone curve,” which is typically defined to be a time-varying, piecewise differentiable curve in the standard three-dimensional affine Euclidean space E with associated vector space **E**. A parametric representation gives the position of a point identified by a spatial parameter s∈D⊂ℝ at time t∈ℝ as a position vector $p_{b}\left(s,t\right)\in E$ with respect to a specific frame of reference ${ℱ}_{0}$ in E . The differentiability requirement on $p_{b}$ is always *at least* that the first derivative of $p_{b}$ with respect to s exists, is piecewise continuous, and is nowhere equal to zero. This condition guarantees that the curve is rectifiable, or in other words has a measurable arc length (Kreyszig, 1991). The curve changes over time, modeling the motion of the robot, and it is presumed to describe the dominant features of the motion of the robot. Since there is no finite set of coordinates that describes every possible curve meeting these requirements, the description of the shape is infinite-dimensional.

The usual type of modeling hypothesis for slender bodies is that other points, which are not located on the backbone, are described by some auxiliary relationship that describes their positions relative to the positions on the backbone. The standard theories from beam mechanics may be adopted for this purpose, in which case the backbone curve may be affixed to the body at the neutral axis of bending^1^. One example is the Euler-Bernoulli hypothesis, which states that sections normal to the backbone remain normal for all deformations. Another is the hypothesis due to Timoshenko stating that normal sections rotate relative to the backbone but remain planar. Standard “warping” theories can be used to couple motion of the points normal to the sections with twisting about the backbone if the sections are not circular. Regardless of these additional hypotheses, the curve is of fundamental importance to the kinematic description.

Explicitly, the body of the robot is identified by the curve through the consideration of a reference configuration $c_{0}$ of the robot. The backbone curve $p_{b}$ is placed on this reference configuration. The curve is then “framed” by a set of unit vectors $d_{1}\left(s,t\right),\;d_{2}\left(s,t\right),$ and $d_{3}\left(s,t\right)$ termed the director vectors. The first two are chosen to be orthogonal and to span the section of the body at s which is normal to the curve. The third is taken to complete a right-handed, orthonormal coordinate frame as $d_{3}\left(s,t\right)=d_{1}\left(s,t\right)\times d_{2}\left(s,t\right)$ . In terms of classical differential geometry, $d_{3}$ is the tangent vector, and $d_{1}$ and $d_{2}$ could be selected as the normal and bi-normal vectors from Frenet’s formulas (Kreyszig, 1991). This procedure is problematic for general curves since torsion may be undefined, but many other alternative framings of the curve are possible which do not suffer this problem (Bishop, 1975). The backbone position and unit vectors together describe a local reference frame ℱ(s,t) for each point along the curve. The unit vectors equivalently define a spatiotemporal field of rotation operators $R_{b}\left(s,t\right)\in SO\left(3\right)$ . The rotation field can be represented by matrices (Rucker and Webster, 2011), quaternions (Boyer et al., 2020), or any other suitable representation. Together with the position vector, a spatiotemporal field of transformations $g_{b}\left(s,t\right)\in SE\left(3\right)$ is defined by. $g_{b}\left(s,t\right)=\left\{R_{b}\left(s,\;t\right),\;p_{b}\left(s,t\right)\right\}.$

The vectors $u\left(s,t\right)=u_{k}d_{k}$ and $v\left(s,t\right)=v_{k}d_{k}$ are termed the “strain variables.” They describe deformation of the body and are invariant under rigid transformations. The vector u has been widely called the “curvature” vector in the robotics literature, but this may be misleading since it is not generally the curvature of the deformed backbone curve. The term “flexural strain” is preferred for $u_{1}$ and $u_{2}$ , and “torsional strain” for $u_{3}$ . The variables $v_{1}$ and $v_{2}$ are called the shear strains, and $v_{3}$ is the dilation. The change in length or “extension” of the backbone curve is characterized by $\partial_{s}p^{2}=v\cdot v$ . The strain variables are related to the framed curve by the following relationships.

$$\begin{array}{c} \partial_{s}p_{b}\left(s,t\right)=v\left(s,t\right),\;\;\;\;\;\partial_{s}d_{k}\left(s,t\right)=u\left(s,t\right)\times d_{k}\left(s,t\right) \end{array}.$$ (1)

Finally, the vectors $\partial_{t}p_{b}\left(s,t\right)$ and ω(s,t) represent the linear and angular velocity of the backbone curve and director vectors. The angular velocity satisfies the equation $\partial_{t}d_{k}\left(s,t\right)=\omega \left(s,t\right)\times d_{k}\left(s,t\right)$ . The four functions $u,v,\partial_{t}p_{b},$ and ω are not independent; they must satisfy $\partial_{s}\omega =\partial_{t}u+u\times \omega$ . In the reference configuration, the flexure strains have non-zero values $u_{0}\left(s\right)$ if the backbone is not a straight. Generally, $v_{0}\left(s\right)=d_{3}\left(s\right)$ , but other choices are possible.

#### The General Continuum Description

The second approach to describing the configuration of continuum robots is to make as few prior kinematic hypotheses on the configuration as possible. The traditional description of a three-dimensional continuum in solid mechanics is used in this case. In this approach a reference configuration $c_{0}$ is identified by their position vector relative to a frame of reference ${ℱ}_{0}$ . Three coordinates $X\in {\mathbb{R}}^{3}$ identify the position of each point in the body via a one-to-one, differentiable vector-valued function P(X) . If X is chosen as the Cartesian coordinates with respect to ${ℱ}_{0}$ , then this function and its inverse are trivial. The final locations of the points are described by p(X,t) . In some cases, it is useful to define a displacement field U as follows.

p(X, t)=P(X)+U(X, t).

The amount of stretching can be quantified by the deformation gradient, defined by

$$F\left(X,t\right)={\frac{\partial p}{\partial P}|}_{X,t}.$$

The deformation gradient straightforwardly describes the local changes in length (amount of stretching) and therefore plays a major role in the definition of strain measures. Note also that the curve-based description of the configuration, together with the classical Euler-Bernoulli hypothesis, can be placed into this more general framework using $X=\left(s,X_{2},X_{3}\right)$ and $p\left(X,t\right)=p_{b}\left(s,\;t\right)+X_{2}d_{2}\left(s,t\right)+X_{3}d_{3}\left(s,t\right)$ (Antman, 2005).

### Perspective on Discretization and Configuration Spaces

There are two perspectives that one might take when describing the kinematics or mechanics of continua. In the first perspective, the model consists of a (possibly nonlinear) PDE, a domain on which the PDE applies, and boundary conditions in the form of constraints or measurements. The robot’s state space consists of the dependent variables related by the PDE. The state space is therefore a particular Cartesian product space that might involve, in general, both finite-dimensional spaces and infinite-dimensional function spaces. In the process of computing a numerical solution to a model, any part of the state that belongs to an infinite-dimensional space must be approximated by a finite set of coordinates in ${\mathbb{R}}^{n}$ , but the choice of coordinates does not need to be of great concern to the modeler. This perspective has been taken by numerous authors for general continuum manipulators (Trivedi et al., 2008; Till et al., 2019), concentric tube robots (Dupont et al., 2010; Rucker et al., 2010; Gilbert et al., 2016), parallel continuum robots (Black et al., 2018), and bioinspired locomotion by snakes and worms (Boyer et al., 2012). The modeler hopes that any approximation error is small enough to be ignored, and error-controlled numerical methods may provide some assurances. This first perspective is the natural one if, for example, the modeler selects an error-controlled, automatic step-size numerical integrator like the Dormand-Prince Runge Kutta pair to approximate the solution to a differential equation with a spatially distributed independent variable. The benefit to this perspective is that questions of convergence may generally be avoided. However, there are two main disadvantages: first, there is a relative paucity of tools available if the problem is not expressed with respect to a single independent spatial variable; second, the degrees of freedom chosen by automatic numerical methods may be unknowable in advance and may vary between model solutions, making it difficult to apply algorithms built on spaces like ${\mathbb{R}}^{n}$ or on manifolds where coordinate charts are available.

In a second perspective, the equations of an infinite-dimensional model are explicitly discretized through a suitable method such as the finite element method or a finite difference method (Renda et al., 2014; Back et al., 2015; Gilbert and Godage, 2019) or via a spectral method involving a “modal” decomposition (Chirikjian and Burdick, 1994; Godage et al., 2015; Chen et al., 2020). In this perspective, the modeler takes control over the discretization and fixes the dimensionality of the resulting model. One is free to take the perspective that a *new* model has been created that is not necessarily subordinate in any way to the infinite-dimensional model. In other words, the infinite dimensional dependent variables, ODEs, and/or PDEs, were only a steppingstone to the finite-dimensional model. The dimension may be varied according to a model hyper-parameter N , and often one wishes that as N→∞ , the solutions to the sequence of fixed-dimensional models approach the solution to a corresponding infinite-dimensional model.

The second perspective is the standard one in generally accepted theories of robot kinematics and dynamics, in which the goal is to find a suitable coordinate set that describes the displacement field u(X,t) that takes a material point located at initial position P to its final position p=P+u . With rigid link manipulators, the space is partitioned into non-intersecting bodies indexed by number $i\in {\mathbb{Z}}^{+}$ and equipped with local coordinate frames, and then the machinery of SE(3) is used to associate each body with its own displacement field expressed in terms of one of the coordinate transformations Ti0∈SE(3) representing the transformation between the base frame 0 and the frame of the $i^{th}$ body. For serial, rigid-link robots, the choice of finite dimensional coordinates parameterizing the displacement field is usually one of two conventions, the Denavit-Hartenberg convention (Denavit and Hartenberg, 1955) or the twist coordinate system and product-of-exponentials formula (Brockett, 1984).

For continuum and soft robots, neither the perspective (finite vs. infinite-dimensional) nor the approach to discretization (choice of coordinates) appears to be standardized. In some cases, restrictive assumptions do allow a set of finite coordinates that uniquely specify the configuration of a continuum robot. For example, Bretl and McCarthy showed that for the Kirchhoff rod with no external loading, a configuration space isomorphic to ${\mathbb{R}}^{6}$ can be selected, corresponding physically to a known internal force and moment at the same location in space as the known orientation of the rod (Bretl and McCarthy, 2014). A similar result is known for coordinates of the configuration space of concentric tube robots without any external loads (Gilbert et al., 2016). The general principle is a basic result on initial value problems. If the mechanics of the system can be modeled by a system of n first-order initial value problems,

$$\partial_{s}y=F\left(s,y\right),\;\;y\left(s_{0},t\right)=y_{0}\left(t\right),$$

with F uniformly Lipschitz in y and continuous in s , then the solutions are uniquely determined by $y_{0}$ (Schaeffer and Cain, 2016). Therefore, if all state information of the robot is contained in the functions $y_{i}\left(s,\;t\right)$ , then it is clear that $y_{0}$ is a suitable set of coordinates for the configuration space of the robot. For curve-based models $y_{0}$ usually belongs to a space of the form $SE{\left(3\right)}^{r}\times {\mathbb{R}}^{n}$ .

However, with less restrictive assumptions, low-dimensional configuration spaces are not generally found. Such is the case for parallel continuum robots (Black et al., 2018), for growing robots (Greer et al., 2019), or soft robotic hands (Schlagenhauf et al., 2018). It is in general impossible to find a “minimal” set of coordinates for the C-space of any continuum manipulator when the locations and nature of external loads or contacts are a-priori unknown and when these loads cause substantial changes in the robot shape. The subsections that follow describe a variety of methods that have been used to mathematically represent the configurations of continuum robots.

#### Spectral Methods

Spectral methods were some of the earliest described methods for the kinematic modeling of backbone curves. In this method, the configuration is represented by a finite number of coordinates $q\left(t\right)\in {\mathbb{R}}^{N}$ by assuming that some kinematic quantity is described by a truncated sum of “modal” shape functions $\phi_{i}\left(s\right)$ in a manner analogous to a Fourier series. The general form is to have a scalar quantity $S_{j}$ represented as

$$S_{j}\left(s,t\right)=\overset{N}{\underset{i=1}{\sum }}a_{ij}\left(q\left(t\right)\right)\phi_{i}\left(s\right).$$

The function $a_{ij}$ may be simply an index into the vector q pulling out one of the components, or it may be a more complicated relationship. The mode shapes are generally selected among one of the standard families such as trigonometric functions $\sin\left(k_{i}\pi s\right)$ and $\cos\left(k_{i}\pi s\right)$ for a series of values $k_{i}\in \mathbb{R}$ (directly analogous to a truncated Fourier series), the standard monomials $\left\{1,s,s^{2},\ldots \right\}$ , the Legendre polynomials, Chebyshev polynomials, etc. (Chirikjian and Burdick, 1994; Zhang and Simaan, 2013; Chen et al., 2020). In general, to be classified as a spectral method, the mode functions should have global support rather than local support, which leads to the element-based methods described below.

There is a great deal of freedom within this approach. For example, the tangent vector $d_{3}$ can be expressed in spherical angles θ(s,t) and ϕ(s, t) , and then $S_{1}=\theta$ and $S_{2}=\phi$ , and v=(0,0,1) completes the kinematic description (Chirikjian and Burdick, 1994). $S_{j}$ could also be chosen directly as a component of the displacement field of the backbone curve (Godage et al., 2015). These methods are extrinsic because they seek to approximate kinematic quantities as measured by the observer in the inertial frame ${ℱ}_{0}$ . Parameterizations also possible which represent the strain variables u(s,t) and v(s,t) measured by an observer in the local frame ℱ(s,t) (Boyer et al., 2020). When coupled with a collocation method used to determine u **,** it was shown that the Magnus expansion can be used to efficiently recover the position and orientation field (Orekhov and Simaan, 2020).

In the context of continuum robots, to the best of the author’s knowledge, the spectral methods have only been applied in conjunction with the curve-based descriptions discussed in *The Curve-Based Description* and not for more general continuum descriptions.

#### Element-Based Methods: PCC

The element-based methods, in contrast to the spectral methods, break up the problem spatially into adjacent sub-domains and attempt to model the kinematics on each sub-domain using a simpler hypothesis. This procedure can be carried out for both the curve-based description and the general continuum description. Many authors have adopted the kinematic hypothesis that the backbone curve is a sequence of circular arcs which are concatenated by imposing tangency conditions. There is a natural extension of this idea to piecewise helical curves. This approximation is termed the “piecewise constant curvature” (PCC) method, and many continuum robots have even been designed to exhibit deformation of this kind, at least in the absence of external loads (Webster and Jones, 2010). For example, multi-backbone robots and tendon-driven robots will adopt, with actuation, shapes very close to circular arcs with appropriate design decisions (Camarillo et al., 2008; Xu and Simaan 2008). On the other hand, even gravitational loading may cause more flexible robots to adopt shapes more complex than a single circular arc (Trivedi et al., 2008).

Within the curve-based framework described above, the “standard” PCC hypothesis including inextensibility and shear-lessness is equivalent to a partitioning of the domain into m elements ${\text{Γ}}_{e}=\left[s_{e-1},\;s_{e}\right]$ with $D=\cup_{e=1}^{m}\;{\text{Γ}}_{e}$ and an approximation of the flexural-torsional strain as the following sum:

$$u\left(s,t\right)=\overset{m}{\underset{e=1}{\sum }}u_{e}\left(t\right)\;{\text{χ}}_{{\text{Γ}}_{e}}\left(s\right)\;,\;\;\chi_{{\text{Γ}}_{e}}\left(s\right)=\{\begin{array}{cc} 1 & s\in {\text{Γ}}_{e}\\ 0 & s\notin {\text{Γ}}_{e} \end{array}v\left(s,t\right)=d_{3}\left(s\right).$$

A similar approach is called the piecewise constant strain (PCS) method and extends the definition to include an approximating sum representing v (Renda et al., 2016). The vectors are resolved in Cartesian coordinates of the frame ℱ(s,t) and it is these body-frame coordinates that are presumed constant over the element.

The major advantage of the PCC method is that the extrinsic variables $p_{b}$ and $d_{k}$ (equivalently $R_{b}$ ) are easily computed using an explicit recursion presuming that a single boundary condition on the pose of the curve, $g\left(s_{0},\;t\right)=g_{0}\left(t\right)$ , is known. On element ${\text{Γ}}_{i}$ , the transformation from ${ℱ}_{0}$ to ℱ(s,t) is given by

g(s, t)=g(se−1, t)exp((s−se−1)ξe(t)),ξe(t)=[ue(t)×ve(t)00]ℱ(s,t).

The mapping exp:se(3)→SE(3) is continuous and differentiable, including at the element 0∈se(3) **,** and it may be computed in closed form, below in vector form but originally discovered by Euler and Rodrigues (Rodrigues, 1840; Cheng and Gupta, 1989).

$$\exp\left(\left[\begin{array}{cc} \omega_{\times } & v\\ 0 & 0 \end{array}\right]\right)=\left[\begin{array}{cc} R\left(\omega \right) & p\left(\omega ,\;v\right)\\ 0 & 1 \end{array}\right]\;R\left(\omega \right)=I+C\left(\omega \right)\omega_{\times }+A\left(\omega \right)\omega_{\times }^{2}\;\;p\left(\omega ,\;v\right)=C\left(\omega \right)v+A\left(\omega \right)\left(\omega \times v\right)+B\left(\omega \right)\left(\omega \cdot v\right)\omega .$$

The coefficient functions are as follows, with sinc(x) the un-normalized sinc function, which is available in the standard libraries of many programming languages.

$$A\left(\omega \right)=\frac{1}{2}{\text{sinc}}^{2}\left(\frac{\left\|\omega \right\|}{2}\right),\;\;B\left(\omega \right)=\frac{\left\|\omega \right\|-\sin\left(\|\omega \|\right)}{{\left\|\omega \right\|}^{3}},\;\;C\left(\omega \right)=\text{sinc}\left(\|\omega \|\right).$$

In IEEE-754 double-precision arithmetic, the author has found that B(ω) may be accurately computed by a truncated Taylor series $B\left(\omega \right)\approx 1/6-{\left\|\omega \right\|}^{2}/120+{\left\|\omega \right\|}^{4}/5040$ if $\left\|\omega \right\|<1\times 10^{-4}$ .

**FIGURE 3:**
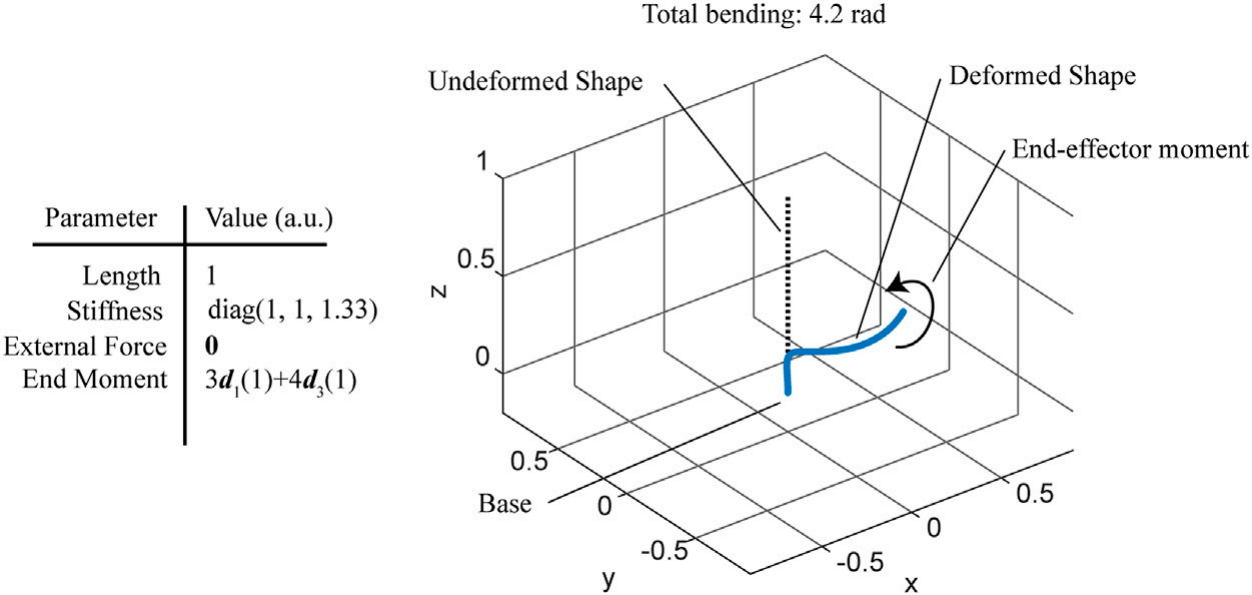
Simulation of a cantilevered rod under a combined bending and twisting concentrated moment, forming a helix.

**FIGURE 4:**
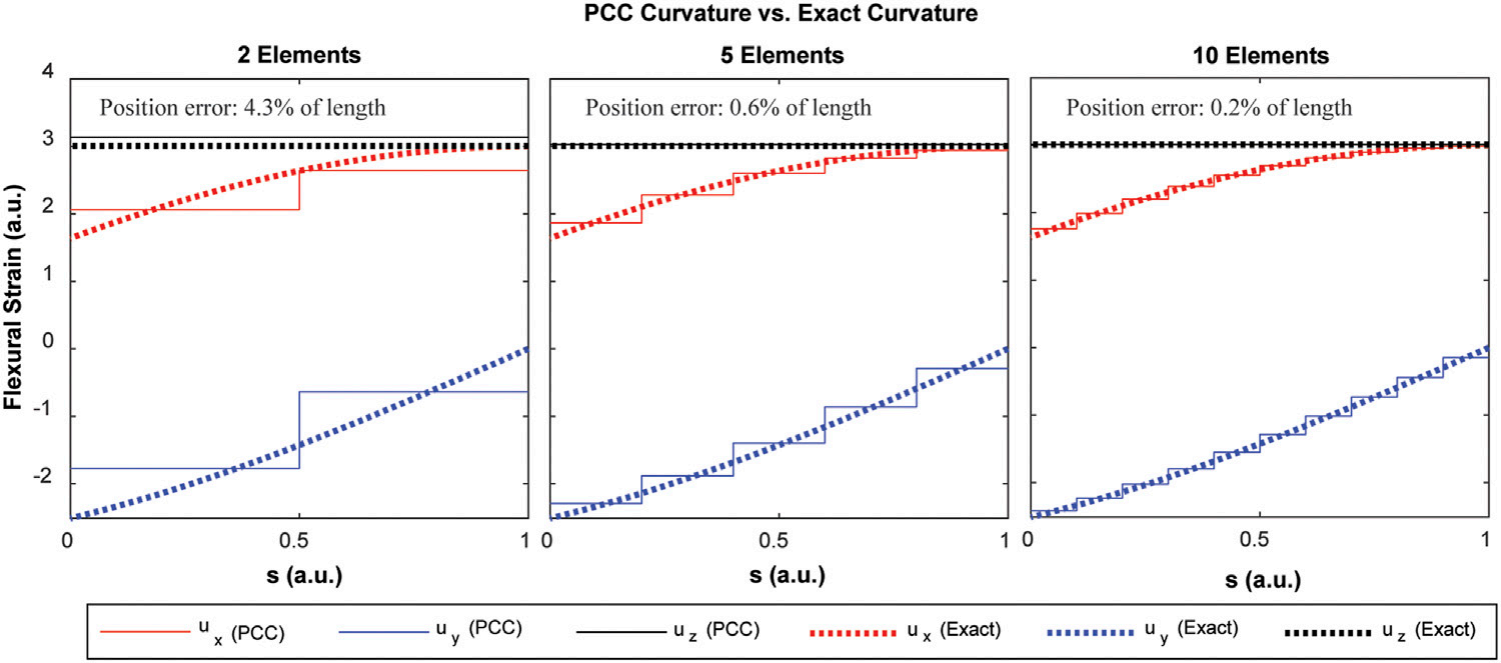
Convergence of the PCC discretization to the exact flexural strains of the helical rod shape depicted in Figure 3. Note that the exact flexural strain components are not constant functions of arc length.

The PCC and/or PCS methods are the simplest explicit and consistent discretization methods for a framed curve which interpolate the intrinsic (strain) variables rather than the extrinsic (position and orientation) variables. In other words, given a curve with bounded flexural, torsional, shear, and extensional strains, the error between the curve and its PCC or PCS approximation (both in terms of $d_{k}$ and in terms of $p_{b})$ shrinks as the number of elements increases. This property is important because it means that the PCC/PCS framework can describe robots with practically any backbone curve if the domain is broken into enough elements. Figure 3 depicts a single isotropic rod under a combined twisting and bending moment, resulting in a helical shape. The results of a PCC approximation under a linear elastic material law and subject to the virtual work principle discussed in *Projection via D’Alembert’s Principle* below are shown in Figure 4. The flexural strain variables of the PCC model approach those of the exact solution as the number of elements increases. The example also demonstrates an important distinction between material flexural strain and the usual notion of *curvature* of a shape. The exact solution under the end load depicted in Figure 3 is indeed a helix, which is a “constant curvature” shape, yet the flexural strain variables, when resolved in components of the material frame ℱ(s,t) or any fixed frame of reference, are not constant functions of the arc length. For this reason and others discussed in *Considerations for Kinematic Hypotheses*, the simplicity of the PCC approach relative to others may not outweigh the drawbacks.

#### Element-Based Methods: Higher-Order One-Dimensional

The discretization of the curve into elements can also be accomplished with higher-order schemes than the piecewise constant strain approach. In general, as with the PCC approach if a boundary condition $g\left(s_{0},\;t\right)$ is provided at a single location along the backbone curve, then the C-space can be defined by approximating the strain variables on each element with an expansion having more terms than the PCS approach (Boyer et al., 2020).

[u(s,t)v(s,t)]ℱ(s,t)=∪e=1m∑j=1Neϕj(ze)qej(t),     ze=s−sese−se−1.

The union operator is abused to mean here that the element-local terms contained within each argument of the union are “assigned” to provide the evaluation on that element. The coordinate $z_{e}$ is a normalized element-local length coordinate identifying the cross section within the element. If the model is to contain flexure, torsion, shear, and extension, then a polynomial expansion of order p in each component entails $N_{e}=6\left(p+1\right)$ degrees of freedom within each element. Continuity conditions at the element boundaries may and/or boundary conditions may reduce the number of total degrees of freedom from $mN_{e}$ as implied by the formula.

Other representations for the C-space based on segmentation into elements are possible but have not been widely pursued within the robotics community. Certainly, the more widely adopted approach within the finite element and computational mechanics communities has been to make the primary kinematic hypothesis at the level of p and/or $d_{k}$ (Simo and Vu-Quoc, 1986), and this has also been studied in the context of continuum robots (Sadati et al., 2019). This is the approach taken, for example, by the ANSYS simulation software in handling nonlinear beam elements via the BEAM188 and BEAM189 elements. These methods require interpolation on the rotation group SO(3) , and some care is required to ensure that the formulation is invariant to rigid-body transformations (Crisfield and Jelenić, 1999). One major advantage of strain interpolation is that it is frame-invariant directly by construction; the major disadvantage is that the calculation of inertial forces is greatly complicated by the spatial coupling of the degrees of freedom.

#### Element-Based Methods: General Continuum

More general finite-element descriptions have also been used to model soft and continuum robots. In this case, the degrees of freedom q(t) directly interpolate the position field p(X, t) over the three-dimensional domain of the body. Using typical first-order (linear) interpolants, the degrees of freedom are the Cartesian coordinates of the nodes of the mesh that breaks the body into discrete volumes. Direct nodal position discretization using finite elements can be used for closed-loop control using a dimensionality reduction scheme based on projection (Bieze et al., 2018). It has also been shown that high-order FEM models with an order reduction method involving fitting to PCC kinematics is effective (Runge et al., 2017). Finally, it has been demonstrated that general 3D FEM with model order reduction based on the Proper Orthogonal Decomposition can produce models amenable to dynamic closed-loop control (Katzschmann et al., 2019b).

#### Direct Nodal Discretization

Closely related to the element-based methods are those based on direct discretization of the variables. Differential operators in the mechanics can be replaced by their equivalent finite-difference operators to form algebraic equations directly, operating on the values of field variables specified at discrete spatial locations $s_{i}$ for the curve-based approach. A finite difference scheme applied directly to the geometrically exact Cosserat equations has been demonstrated for both the planar case and the spatial case (Hasanzadeh and Janabi-Sharifi, 2014; Gilbert and Godage, 2019; Wang et al., 2021) and described for concentric tube robots (Webster and Rucker, 2009). Finite-difference methods were also used with direct spatial discretization to model a soft underwater arm driven by cables (Renda et al., 2014).

#### Pseudo-Rigid Body Methods

The pseudo-rigid body methods replace the continuum with an approximating rigid linkage. If the curve is broken into a sequence of chords with rotational joints at the nodes joining the chords, then this is equivalent to a spatial “lumping” of the flexural strains into a discrete point via the use of the Dirac delta distribution (Chirikjian and Burdick, 1991; Greigarn et al., 2019).

uℱ(s,t)(s,t)=∑i=1mqi(t)δ(s−si)ni.

A universal joint is the result if two orthogonal axes $n_{i}$ and $n_{i+1}$ are placed with $s_{i}=s_{i+1}$ with both axes normal to the backbone curve. Three orthogonal axes create a spherical joint.

It has been shown that the kinematics of tip-loaded cantilever beams can be modeled adequately by a serial 3R mechanism (Su, 2009). Other PRB models have been created for modeling of catheters (Ganji and Janabi-Sharifi, 2009), tendon-driven continuum manipulators for minimally invasive surgery (Penning and Zinn, 2014), and MRI-actuated catheters (Greigarn et al., 2017). A 6-DOF PRB segment model has also been proposed (Venkiteswaran et al., 2019). An equivalence has also been shown between the coordinates of a PCC model and a suitably defined pseudo-rigid body model, indicating that PRB model segments with RPPR kinematics can be used to describe the same configuration space as PCC models (Katzschmann et al., 2019a).

#### Initial Value Problem Concepts

There are additionally a variety of other methods of analysis and computation which do not explicitly select the degrees of freedom in the kinematic description. In these methods, the unknowns are conceptually left as unknown functions, and numerical methods are used which automatically select the degrees of freedom used to represent the unknown functions, usually via an error estimation and control algorithm.

These methods have been used when the problem is re-cast as a one-dimensional boundary value problem with split boundary conditions.

$$\partial_{s}y=f\left(s,y\right),\;\;G_{a}\left(y\left(0,t\right),\;t_{j}\right)=0,\;\;G_{b}\left(y\left(L,t\right),\;t_{j}\right)=0.$$

Solutions can then be provided by numerical codes which automatically determine the degrees of freedom used to approximate the function $y\left(s,\;t_{j}\right)$ for each discrete value of $t_{j}$ . For continuum robots these methods have been demonstrated via collocation (Webster and Rucker, 2009) and shooting methods (Till et al., 2015; Mauzé et al., 2020) using numerical tools that approximate $y\left(s,\;t_{j}\right)$ via piecewise polynomials. It has also been shown recently that the dynamics problem for a wide variety of architectures based on single or multiple Cosserat rod sub-models can be cast as a shooting problem on an ODE once the time derivatives have been discretized using finite differences (Till et al., 2019).

#### Differential Kinematics for Strain-Variable Hypotheses

It is often necessary to calculate a manipulator “Jacobian field” based on the curve parametrization, and if the generalized coordinates are defined to interpolate the strain variables, this field is not trivial to calculate.

$$\begin{array}{c} \left[\begin{array}{c} \partial_{t}p\\ \omega \end{array}\right]\left(s,t\right)=J_{q}\left(s,t\right)\partial_{t}q\left(t\right)=\left[\begin{array}{c} J_{p}\\ J_{\omega } \end{array}\right]\partial_{t}q\left(t\right) \end{array}.$$ (2)

Letting $J_{i}$ be the column multiplied by $\partial_{t}q_{i}\left(t\right)$ , then the column can be calculated from the following differential relationships:

$$\begin{array}{l} \partial_{s}J_{pi}=\partial_{q_{i}}v\\ \partial_{s}J_{\omega i}=\partial_{q_{i}}u+u\times J_{\mathrm{\omega i}}. \end{array}$$

One must take care when the interpolation is carried out on the strain variables in coordinates of the local frame ℱ(s,t) . If desired, the coordinates in the body frame representations Jpiℱ(s,t) and Jωiℱ(s,t) may be calculated from the appropriate representation of these equations in the moving frame (Rucker and Webster, 2011).

∂s (Jpiℱ(s,t))=−uℱ(s,t)×Jpiℱ(s,t)+∂qi(vℱ(s,t))+Jωiℱ(s,t)×vℱ(s,t) ∂s (Jωiℱ(s,t))=−uℱ(s,t)×Jωiℱ(s,t)+∂qi(uℱ(s,t)).

From a known boundary condition where $J_{pi}\left(0,t\right)=0$ and $J_{\omega i}\left(0,t\right)=0$ , the solution to these equations can be expressed in closed form as the solution to a linear time varying system.

[JpiJωi](s)=∫0sexp(−∫0radξ(τ)dτ) ∂q[vℱ(s,t)uℱ(s,t)]dr,  adξ(τ)=[u×v×0u× ]ℱ(s,t).

### Mechanics

Regardless of how the shape of a robot is described, the principles of classical mechanics are frequently used to describe the relationships between the model’s degrees of freedom, the internal stresses, and any imposed boundary conditions which may include external forces, imposed positions or orientations of parts of the robot, contact conditions. The robot’s actuators may generally be modeled in one of two ways: either they are described as constraints (a form of boundary condition) or as sources of internal stress.

#### The Equations of Motion for the Special Theory of Cosserat Rods

In the curve-based description, the equations of motion of the special theory of Cosserat rods serve as the strong form differential equations governing the mechanics (Antman, 2005).

$$\begin{array}{c} \partial_{s}n+f=\rho A\partial_{tt}p+\rho I_{k}\partial_{tt}d_{k} \end{array}$$ (3)

$$\begin{array}{c} \partial_{s}m+\partial_{s}p\times n+\ell =\rho I_{k}d_{k}\times \partial_{tt}p+\partial_{t}\left(\rho J\cdot \omega \right) \end{array}.$$ (4)

The sum from k=1 to 3 is implied over the terms involving $I_{k}$ and $d_{k}$ . The variables n(s,t) and m(s,t) are the internal force and the internal moment, which are interpreted as the resultant force and resultant moment of the stress acting on section s . In the case of a slowly accelerating body, which is typical in many biomedical applications, a quasistatic approximation may be used, in which all terms on the right-hand side are neglected (Burgner-Kahrs et al., 2015). f and ℓ are externally applied forces and moments. Applied concentrated forces and moments require the Dirac δ distribution to express in this formulation.

In the case of a model which allows freedom in all the strain variables, m and n are algebraically related to the kinematic variables through a suitable material constitutive law. On the other hand, in the shear-less and extension-less model, n is a basic unknown and is equivalent to a Lagrange multiplier which enforces the constraint $v\left(s,t\right)=v_{0}\left(s\right)$ .

The parameter ρA is the mass density (expressed per unit length) of the cross-section. ρJ is the mass moment of inertia (per unit length) of the section, which makes ρJ⋅ω the angular momentum (per unit length) calculated about the mass center of the section. The three parameters $\rho I_{k}$ account for linear momentum density of the cross section caused by angular velocity of the curve. The author is not aware of any works in the robotics literature for which this term has been nonzero; if the backbone curve is chosen to pass through the mass centers of the cross sections, then $\rho I_{k}=0$ and the equations simplify considerably. However, it is noteworthy that this may in general result in the curve failing to pass through the cross-section centroids (if multiple materials are used) or it may be impossible to satisfy this requirement exactly if a single curve is used to model a body with complex geometric features.

#### The Equations of Motion for Pseudo-Rigid Body Models

With the PRB-type models, the equations of motion are exactly those of a classical multibody dynamical system with scleronomous, holonomic constraints. These equations are commonly given as follows (Murray et al., 2017).

$$M\left(q\right)\ddot{q}+C\left(q,\dot{q}\right)\dot{q}+N\left(q,\dot{q}\right)=B\left(\tau \right).$$

The right-hand side contains the non-conservative generalized forces associated with actuation and any other forces; since the robots are underactuated there are generally many more rows in this equation than actuator variables $\tau_{i}$ . Also, it is noteworthy that the inertial forces are not trivial to calculate since the motion of the continuum body is not the same as the motion of the rigid-link approximation. Some assumptions about how the continuum “tracks” the rigid-link approximation as it moves is needed. One approach is to match the centers of mass of chords along the curves of a PCC model with centers of mass of the links in the rigid link model (Della Santina et al., 2018).

#### The Equations of Motion for General Deformable Bodies

The dynamic equilibrium conditions of classical continuum mechanics serve as the defining relationship for general three-dimensional finite element models of soft and continuum robots. Rarely are these equations encountered explicitly in the literature on continuum robots, with most authors preferring to state the result after the strong form equations have been converted to the weak form and integrated. The resulting equations, incorporating constraint forces, are of the following form (Goury et al., 2021).

$$M\left(q\right)\ddot{q}+F\left(q,\dot{q}\right)+G\left(q\right)=H^{T}\lambda$$

The form of this equation is directly analogous to the classical form of the dynamical equations for rigid multibody systems. $M\left(q\right)\ddot{q}$ accounts for the inertial forces, $F\left(q,\dot{q}\right)$ accounts for the internal forces produced by deformation of the material, and G(q) accounts for gravitational forces. The matrix H is associated with the constraints and boundary conditions and encodes the effect of the boundary and actuation forces contained in the vector λ . The details of the construction procedure for this equation are out of the scope of this paper.

#### Projection via D’Alembert’s Principle

In the case of the curve-based models using either the PCS or higher-order models, the equations can be projected onto the degrees of freedom of the model using Galerkin’s principle, probably better known among mechanical engineers as the principle of virtual work (Greenwood, 1988). The method is also equivalent in results to Kane’s method of virtual power (Kane and Levinson, 1983; Rone and Ben-Tzvi, 2014). Because the backbone curve descriptions for the PCC, PCS, and higher order strain variable interpolants are described by independent degrees of freedom $q\in {\mathbb{R}}^{N}$ , a direct projection of the equilibrium equations is possible via D’Alembert’s principle, which amounts to an integration over the equations of motion.

$$\begin{array}{l} \begin{array}{c} \begin{array}{c} \overset{L}{\underset{0}{\int }}\left[\left(F\left(s,t\right)+F^{*}\left(s,t\right)\right)\cdot \gamma_{j}\left(s,t\right)+\left(M\left(s,t\right)+M^{*}\left(s,t\right)\right)\cdot \beta_{j}\left(s,t\right)\right]\;ds=Q_{j,nc} \end{array}\\ j=1,\ldots ,N \end{array}\\ \begin{array}{cc} F\left(s,t\right)=-\partial_{s}n\left(s,t\right), & F^{*}\left(s,t\right)=\rho A\partial_{tt}p\left(s,t\right)+\rho I_{k}\partial_{tt}d_{k}\left(s,t\right)\\ M\left(s,t\right)=-\partial_{s}m+\partial_{s}p\times n,\; & M^{*}\left(s,t\right)=\rho I_{k}d_{k}\times \partial_{tt}p+\partial_{t}\left(\rho J\cdot \omega \right) \end{array}\\ Q_{j,nc}=\overset{L}{\underset{0}{\int }}f\left(s,t\right)\cdot \gamma_{j}\left(s,t\right)+\ell \left(s,t\right)\cdot \beta_{j}\left(s,t\right)\;\text{d}\tau . \end{array}$$ (5)

The velocity coefficient function and angular velocity coefficient function are defined as

$$\gamma_{j}\left(s,t\right)=\partial_{q_{j}}p\left(s,t\right)=\partial_{{\dot{q}}_{j}}\partial_{t}p,\;\beta_{j}\left(s,t\right)=\partial_{{\dot{q}}_{j}}\omega .$$

The velocity coefficients are the “Jacobian field” satisfying the relation Eq. 2.

Since the time derivatives of the momentum density and angular momentum density, $\partial_{tt}p$ and $\partial_{t}\left(\rho J\cdot \omega \right)$ , can be written as linear functions of the $\partial_{tt}q$ , the equations of motion are linear in the accelerations of the generalized coordinates, as expected. In the case of the PCC/PCS kinematic description, the derivatives $\partial_{s}n$ and $\partial_{s}m$ **,** if resolved in ℱ(s,t) , are zero except at the element boundaries. The equations may be integrated by parts into a form which shows the conjugacy of n and v and the conjugacy of m and u .

$$\begin{array}{l} \overset{L}{\underset{0}{\int }}\partial_{s}n\cdot \gamma_{j}\;ds={\left[n\cdot \gamma_{j}\right]}_{0}^{L}-\overset{L}{\underset{0}{\int }}n\cdot \partial_{q_{j}}v\;\text{d}s\\ \overset{L}{\underset{0}{\int }}\partial_{s}m\cdot \beta_{j}\text{d}s={\left[m\cdot \beta_{j}\right]}_{0}^{L}-\overset{L}{\underset{0}{\int }}m\cdot \left(\partial_{q_{j}}u+u\times \beta_{j}\right)\text{d}s. \end{array}$$

In the local frame, the equations take the following forms.

∫0L∂sn⋅γj ds=[nℱ(s,t)⋅γjℱ(s,t)]0L−∫0Lnℱ(s,t)⋅(∂qj(vℱ(s,t))+βjℱ(s,t)×vℱ(s,t))ds∫0L∂sm⋅βjds=[mℱ(s,t)⋅βjℱ(s,t)]0L−∫0Lmℱ(s,t)⋅∂qj(uℱ(s,t)) ds.

Note also that if n(s,t) is constant over s , the first integral is trivially zero.

Finally, note that if more than one rod-like body is present, then a sum over the bodies takes place in Eq. 5. Explicit constraints between the bodies may be handled via the method of Lagrange multipliers.

#### Learning-Based Approaches

Learning-based approaches, which are also sometimes referred to as “model-free” approaches, may be able to describe the relationships between the actuator inputs and observable outputs such as the end-effector motion without recourse to physical parameters and the laws of mechanics. These models usually serve a complementary purpose to those based on physical first principles. Since they require training data from a real robot or from another simulation model, they may be used for on-line control, inverse and forward kinematics, or for off-line analysis and testing of other algorithms such as for navigation and control. The a-priori prediction of behaviors from only design data is generally not possible to date using only learning-based methods.

A variety of purely kinematic approaches have been proposed. One learning approach uses an on-line estimation of the Jacobian matrix relating the time derivatives of the actuation variables $\partial_{t}\tau$ to the time derivatives $\partial_{t}p\left(L,t\right)$ and $\partial_{t}d_{k}\left(L,t\right)$ , and it has been shown that this approach works for both position control and hybrid position/force control when appropriate sensing is available in hardware (Yip and Camarillo, 2014; Yip and Camarillo, 2016). Since the method requires no information about the robot or the environment a-priori, it enables control in complex scenarios, where highly complex physics-based models may have poorly observable parameters or states. It has also been shown that inverse kinematics for continuum robots may be approximated by a multilayer perceptron network (George et al., 2017; Grassmann et al., 2018; Lai et al., 2019), with multi-agent reinforcement learning (Ansari et al., 2016), with K-nearest neighbors and Gaussian mixture regression (Chen and Lau, 2016), and with deep reinforcement learning (Satheeshbabu et al., 2019). For reconfigurable robots subject to varying loads, it has been shown that classification of the load state using long short-term memory networks can substantially improve open-loop kinematic control (Nicolai et al., 2020). For flexible catheters, a combination of a support vector machine classifier and a fully connect neural network regressor were demonstrated achieving sub-millimeter trajectory tracking errors (Jolaei et al., 2020). Learning may also play an important role in proprioception for continuum robots with distributed sensing, where additional sensors beyond actuator-collocated ones are available for measuring the robot shape (Truby et al.,2020).

It has also been shown that dynamic models may be learned. Under a state observation of the form x=F(q) provided by sensors, where it is presumed that the dimension of x and q are the same and that F is invertible, the dynamics of the system can be posed as a one-to-one mapping $\left(\tau ,x,\partial_{t}x\right)\to \partial_{tt}x$ . On a real or simulated robot, this map representing the dynamics of the observables of the system can be approximated in discrete time via recurrent neural network (Thuruthel et al., 2017). Note that the kinematic relationships under static conditions are obviously also contained in this trained map for all points satisfying (τ,x,0)→0 . A similar approach using deep neural networks has also been demonstrated (Gillespie et al., 2016). Data-driven system identification based on the Koopman operator theory has led to control-oriented dynamic models amenable to model-predictive control (Bruder et al., 2020). Autoregressive with exogenous input (ARX) and nonlinear autoregressive with exogenous input (NARX) models have been studied for a single-section tendon-driven continuum robot, with the conclusion that NARX provides advantages in modeled end-effector position accuracy (Parvaresh and Moosavian, 2019). For closed-loop dynamic control, the direct reinforcement learning of a control policy which learns the mapping from current robot states, previous robot states, and desired end-effector position to the appropriate control action is possible (Thuruthel et al., 2019).

There are also learning-based approaches to control which do not explicitly construct kinematic or dynamic models. One such approach is based on direct learning from demonstration in the actuator space, which was successfully demonstrated on a tendon-driven continuum manipulator (Xu et al., 2016). Learning can form a part of a.

#### Actuator Models

Actuators in continuum and soft robots have been classified as either extrinsic, in which case the actuators are not a part of the deformable body, or intrinsic, in which case the actuators are an integral part of the deformable body. Examples of the former include tendons, the boundary conditions placed on concentric tube robots. Examples of the latter include soft pneumatic muscles (Walker et al., 2016).

The actuators may be modeled (very generally) as relationships between the actuation variables, generalized forces, and the dynamic state of the robot consisting of ***q*** and $\partial_{t}q$ .

$$G_{i}\left(\tau_{i},\;q,\partial_{t}q,\;Q_{nc}\right)=0.$$

However, the nature of the model may change depending on the exact form of $G_{i}$ . If $G_{i}$ involves only $\tau_{i}$ and q , then it is exactly in the form of a holonomic constraint. In general, it may not be simple to find a reduced set of independent coordinates satisfying the constraint, and a Lagrange multiplier technique may be required to enforce it. On the other hand, if $G_{i}$ can be inverted to find $Q_{nc}={\hat{G}}_{i}\left(\tau_{i},\;q,\;\partial_{t}q\right)$ , then the actuation can be directly coupled to the equations of motion. Which of these two views of actuation is the more natural one depends on the characteristics of the particular actuator(s) and sensor(s) chosen.

A first example is the model of a fiber-reinforced elastic actuator, in which V is the enclosed fluid volume of the actuator, τ=P is the fluid pressure, and $J_{V}$ is the Jacobian matrix relating the changes in the generalized coordinates to the change in volume of the fluid (Sedal et al., 2021). Then, the principle of virtual work indicates that

$$\delta W=\tau \;\delta V=\tau \;J_{v}\left(q\right)\delta q=Q_{i,nc}\delta q_{i}$$

$$Q_{nc}=J_{V}^{T}\left(q\right)\tau ,\;J_{V}\left(q\right)=\partial_{q}V.$$

Another explicit example is found in the case of a tendon-driven robot. If enough support for the tendon is provided, a reasonable model for the points occupied by the tendon is a continuous curve described by $p_{t}\left(s,t\right)=p\left(s,t\right)+a\left(s\right)$ with $a\left(s\right)=a_{1}\left(s\right)d_{1}\left(s,t\right)+a_{2}\left(s\right)d_{2}\left(s,t\right)$ (Rucker and Webster, 2011). For the sake of simplicity, restrict the tendon to a planar path with $a_{2}\left(s\right)=0$ . The tendon length can then be calculated as an integral functional involving the deformation gradient evaluated along the tendon path using the curve-based kinematic hypotheses:

$$\begin{array}{c} \ell_{t}\left(q\right)=\int_{0}^{L}d\ell ,\;\;\;d\ell^{2}=ds^{2}\left(d_{3}+\partial_{s}a\right)F_{t}^{T}F_{t}\left(d_{3}+\partial_{s}a\right),\;\;\;\;F_{t}=\frac{\partial p_{t}}{\partial P_{t}}.\; \end{array}$$ (6)

If the tendons are not fully constrained, other models for $\ell_{t}\left(q\right)$ may be more appropriate (Rao et al., 2021). What is noteworthy about either length formulation is that the nonconservative generalized forces do not naturally appear. If the tendon lengths are a known quantity, the actuator model is a holonomic constraint on the generalized coordinates. The problem can be treated via the method of Lagrange multipliers. The Lagrange multiplier will be exactly the tendon tension, and the principle of virtual work can be used to reveal the exact form of the terms in $Q_{nc}$ corresponding to the Lagrange multiplier.

$$\delta W=\tau \delta \ell_{t}=\tau \;J_{\ell }\left(q\right)\;\delta q=Q_{j,nc}\delta q_{j},\;\;J_{\ell }\left(q\right)=\partial_{q}\ell_{t}\left(q\right).$$

Therefore, the effect of the tendon alone (not considering any frictional forces) must be

$$Q_{nc}=J_{\ell }^{T}\left(q\right)\tau .$$

Note that the causal form in which the tendon tensions are known is “easier” to handle since no additional equations must be added. The causal form involving known tendon lengths requires the addition of the nonlinear length constraints Eq. 6 to the equation set and the tension becomes an algebraic unknown along with the accelerations, forming a nonlinear differential-algebraic system in the dynamic case or a nonlinear algebraic system in the quasistatic case. The need to solve a DAE system disappears if the tendon is considered a spring element, since then the force is determined as a function of the difference between $\ell_{t}\left(q\right)$ and the tendon displacement input d .

The resulting model form as a set of ordinary differential equations or differential algebraic equations is shown for a variety of common continuum robot actuators in Table 2.

**TABLE 2:** Model form as an ODE or DAE system based on actuator type, assuming a single rod model architecture for the model.

Actuator input	Model form
Inextensible tendon length	DAE
Extensible tendon length	ODE
Tendon force	ODE
Pneumatic pressure	ODE
Hydraulic pressure (incompressible fluid)	ODE
Hydraulic volume (incompressible fluid)	DAE

### Materials

The kinematic hypotheses and mechanics models must be augmented by constitutive laws (material models) to complete the model of a continuum robot. For quasistatic models, the choice is usually between linear elasticity and other hyperelastic material models. For dynamic models, an additional choice of damping or friction laws is generally required to produce realistic responses.

#### Linear Elasticity

In the case of quasistatic models, a common assumption in the literature has been to assume a Hookean (linear) material response. In this case, if one assumes that the backbone curve passes through the neutral axis of bending, the following constitutive laws apply:

mℱ(s,t)=Kbtℱ(s,t)(uℱ(s,t)−u0ℱ(s,t)) nℱ(s,t)=Kseℱ(s,t)(vℱ(s,t)−v0ℱ(s,t)).

The matrices $K_{bt}$ and $K_{se}$ may be calculated from the classical Euler-Bernoulli or Timoshenko beam theories and the entries are the flexural and torsional rigidities and shear and extension rigidities, respectively. The explicit relationships follow below (Antman, 2005).

$$m_{\alpha }\left(s,t\right)=\left(EJ_{\alpha \beta }\right)\left(s\right)\left[u_{\beta }\left(s,t\right)-u_{\beta 0}\left(s\right)\right],\;\;m_{3}=D\left(s\right)u_{3}\left(t\right).$$

Note that bending about $d_{1}$ and $d_{2}$ are characterized by the second moments of area and the Young’s modulus E , while the torsional moment is related to the torsional strain variable by a coefficient D solving the St. Venant torsion problem. Only in the case of isotropic rods with circular cross section is this equal to the usual shear modulus G times the polar moment of area $J_{33}$ . Formulas for a wide variety of cross sections that are uniform over s have been tabulated (Roark et al., 2002). The Timoshenko model for shear and elongation adds the following relationships.

$$n_{\alpha }=\left(GA\right)\left(s\right)\;v_{\alpha },\;\;n_{3}=\left(EA\right)\left(s\right)\;\left[v_{3}-1\right].$$

#### Hyperelastic Material Models

Many other hyperelastic models are possible choices, such as Yeoh, neo-Hookian, Gent, Ogden, and Mooney-Rivlin (He et al., 2018; Shiva et al., 2019; Zhang et al., 2019; Antonelli et al., 2020; Bacciocchi and Tarantino, 2021; Zhao et al., 2021). Although in general one may expect that these more complex material models should offer improved model accuracy, it has been shown recently that, at least for some robot designs, a linear stress-strain response may be more than adequate (Shiva et al., 2019). Any hyperelastic law can be represented within the Cosserat rod framework as a strain energy density function W.

W=W(uℱ(s,t),vℱ(s,t))

mℱ(s,t)=∂uW,  nℱ(s,t)=∂vW.

The details of these calculations for each of the respective hyperelastic models is omitted for the sake of brevity and can be found in the cited references.

#### Damping and Friction

The introduction of dissipative mechanisms is generally necessary to encourage numerical stability in dynamic models and to produce realistic dynamic responses. Additionally, in some cases static friction plays a significant role in determining the quasistatic solutions, such as in tendon-driven catheters (Jung et al., 2014). Viscous damping may be introduced via the Kelvin-Voigt material model, which extends the linear elastic models to include rate-dependence in the stress-strain relationship (Gilbert and Godage, 2019; Mustaza et al., 2019).

In the curve-based framework, the Kelvin-Voigt law takes the following form (Linn et al., 2013):

mℱ(s,t)=Kbtℱ(s,t)(uℱ(s,t)−u0ℱ(s,t))+Bbtℱ(s,t) ∂t(uℱ(s,t))nℱ(s,t)=Kseℱ(s,t)(vℱ(s,t)−v0ℱ(s,t))+Bseℱ(s,t) ∂t(vℱ(s,t)).

The matrices $K_{bt}$ and $B_{bt}$ are related by time constants referred to as the extensional retardation time constant, $\tau_{e}=\eta_{E}/E$ , and the shear retardation time constant, $\tau_{s}=\eta /G$ , with $\eta_{E}$ the “extensional viscosity” and η the shear viscosity.

Bbtℱ(s,t)=Kbtℱ(s,t)⋅diag(τe, τe, τs)Bseℱ(s,t)=Kseℱ(s,t)⋅diag(τs, τs, τe).

Static friction models have also been considered for concentric tube robots (Lock and Dupont, 2011), tendon-driven continuum robots (Li et al., 2020), and continuum robots having sheathed tendons or multiple actuated backbones (Roy et al., 2017).

## Discussion

The wide variety of modeling choices that have been described offer the modeler an almost paralyzing array of choices. In the subsections that follow, several questions are posed. The available evidence from the literature as well as analyses guided by classical theories of mechanics are used to discuss these questions and to provide guidance during the initial stages of selecting modeling approaches.

### Considerations for Kinematic Hypotheses

**FIGURE 5:**
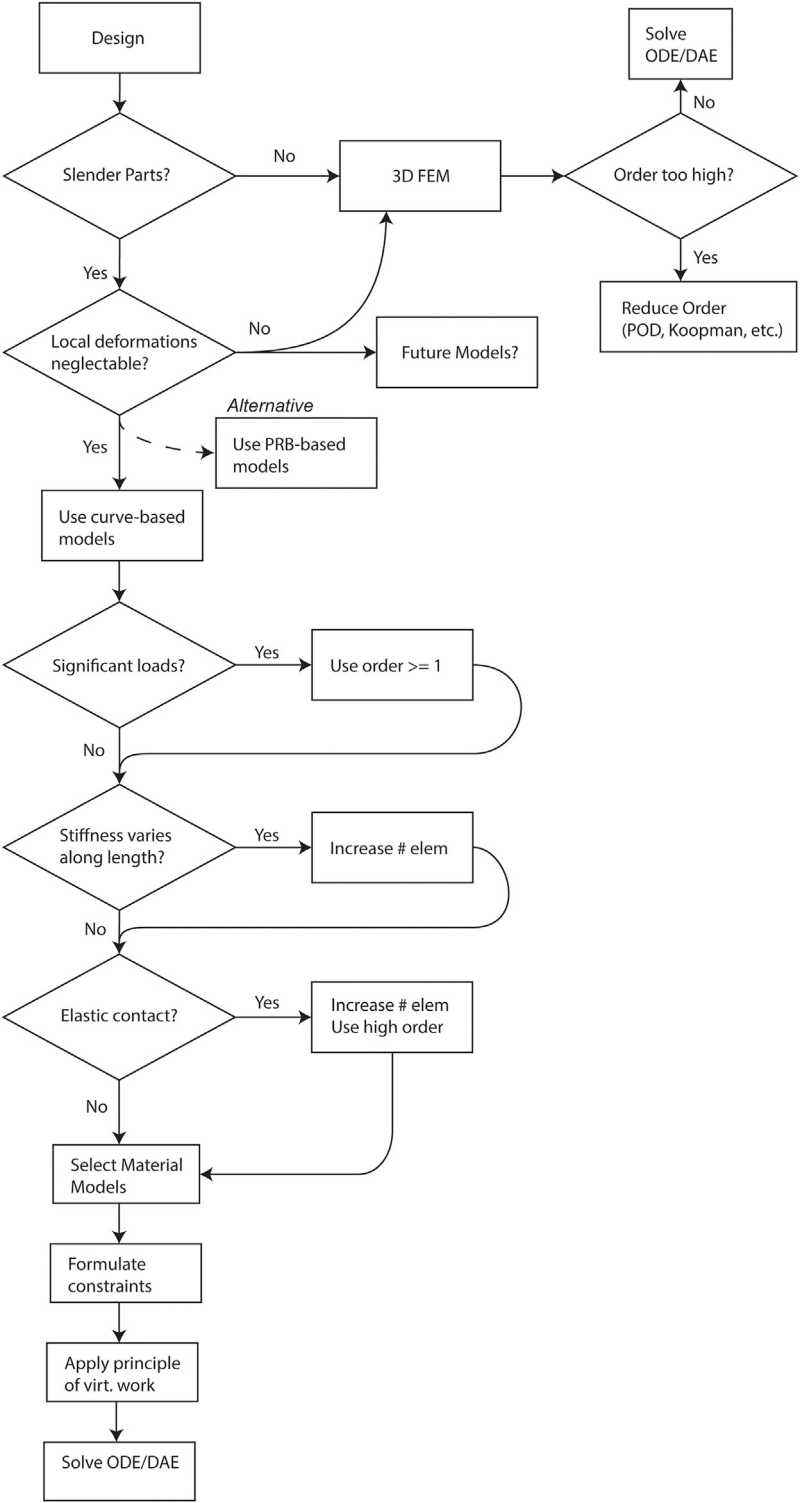
Flowchart depicting the modeling decisions to be made when selecting a model type for a biomedical continuum robot.

The literature on modeling of continuum and soft robots suggests that errors in kinematic models, quantified by the absolute tip positioning error as a percentage of the overall root length, are typically on the order of a few percent. Therefore, there may be little benefit to increasing the order of a spectral method or to further subdividing the domain in an element-based method once the absolute accuracy with respect to the true solution reaches this point. In the sections that follow, analysis and recommendations for kinematic hypotheses which are derived from consideration of the mechanics of bending are offered. Table 3 provides a summary of the recommendations in terms of increasing either the number of elements or the order of the interpolation (assuming that u is the interpolated variable). Figure 5 depicts the decisions leading to the various types of models that have been discussed.

**TABLE 3:** Summary of recommendations to increase either the number of elements or the order of interpolants based on model assumptions and robot-environment conditions.

*Condition/Recommendation*	Number of elements	Element order (curvature interpolant)
Concentrated forces	−	≥1
Non-uniform flexural rigidity	↑	−
Uniformly distributed loads	−	≥2
Elastic contact	↑	↑

#### Considerations for Cantilevered Concentrated Loadings

For continuum robots which are soft enough to exhibit substantial compliance to environmental loads (for example those that may be presented by contact with human anatomy), one of the first considerations for modeling should be consistency with the requirements for accurately modeling cantilevered, concentrated loads.

Let the Cosserat rod equations be recast in terms of the angle of the tangent vector and the load and deformation fixed to the plane defined by $d_{3}\left(0,t\right)$ and $d_{1}\left(0,t\right)$ , let the boundary condition g(0,t) be fixed, and let the load be concentrated at s=L and modeled by $F=P\delta \left(s-L\right)d_{1}\left(0,t\right)$ for the scalar force magnitude P . Furthermore, assume the material response is linear and that the robot is inextensible. These restrictions simplify the problem and result in the following dimensionless boundary value problem governing the angle θ , which represents the tangent vector:

$$d_{3}\left(s,t\right)=\cos\left(\theta \right)d_{3}\left(0,t\right)+\sin\left(\theta \right)d_{1}\left(0,t\right)$$

$$\partial_{\xi \xi }\theta +\lambda \cos\left(\theta \right)=0,\;\;\xi =\frac{x}{L},\;\;\lambda =\frac{PL^{2}}{EI}\;\;\theta \left(0,t\right)=0,\;\;\partial_{\xi }\theta \left(1,t\right)=0.$$

The boundary value problem has a known solution:

$$\theta \left(\xi ,t\right)=2\sin^{-1}\left(k\text{sn}\left(K\left(k\right)-\left(1-\xi \right)\sqrt{\lambda };k^{2}\right)\right)-\frac{\pi }{2}.$$

The quantity k is a scalar that may be found by Newton-Raphson iteration on the following equation, which is implied by the boundary condition θ(0)=0

$$k\text{sn}\left(K\left(k\right)-\sqrt{\lambda };k^{2}\right)=\sqrt{2}/2.$$

The PRB models have the attractive property that they map the problem back into the domain of traditional robotic manipulators, with the obvious advantage that all the tools and knowledge that have been developed in that context (in general, restricted to underactuated mechanisms) now apply to the continuum robot. In the traditional PRB models, the inertia properties are lumped into the links formed by the model, and the stiffness and damping properties are lumped into the joints between links. This lumping introduces error, but it has been shown that optimization of the parameters of the rigid body model can lead to accurate mechanical responses for both cantilevered transverse loads and for applied or internal moments (Chen et al., 2011). Given that the optimal 3R planar PRB model has three degrees of freedom, it is a fair comparison to place the model in competition with other three-DOF models. Here we consider the following three sets of potential kinematic hypotheses and matching constitutive laws and compare them with each other and with the exact solution. Without loss of generality, let L=1 and EI=1 .

Piecewise constant curvature:

$$u_{\text{PCC}}=\sum_{e=1}^{3}u_{e}\;{\text{χ}}_{{\text{Γ}}_{e}}\left(s\right)\;d_{2},\;\;\chi_{{\text{Γ}}_{e}}\left(s\right)=\{\begin{array}{cc} 1 & s\in {\text{Γ}}_{e}\\ 0 & s\notin {\text{Γ}}_{e} \end{array},\;\;D=\left[0,\frac{1}{3}\right]\;\cup \;\left[\frac{1}{3},\frac{2}{3}\right]\;\cup \;\left[\frac{2}{3},1\right]\;m\left(s\right)=EI\;u\left(s\right).$$

*PRB:*

$$u_{\text{PRB}}=\sum_{i=1}^{3}q_{i}\delta \left(s-s_{i}\right)\;d_{2},\;\;s_{1}=0.125,\;\;s_{2}=0.475,\;s_{3}=0.863,\;\;D=\left[0,1\right]\;\frac{m\left(s_{1}\right)}{q_{1}}=3.25,\;\;\frac{m\left(s_{2}\right)}{q_{2}}=2.84,\;\;\frac{m\left(s_{3}\right)}{q_{3}}=2.95.$$

*Spectral:*

$$u_{\text{S}}=\sum_{i=1}^{3}q_{i}s^{i-1}\;d_{2},\;\;D=\left[0,1\right].$$

m(s)=EI u(s)

**FIGURE 6:**
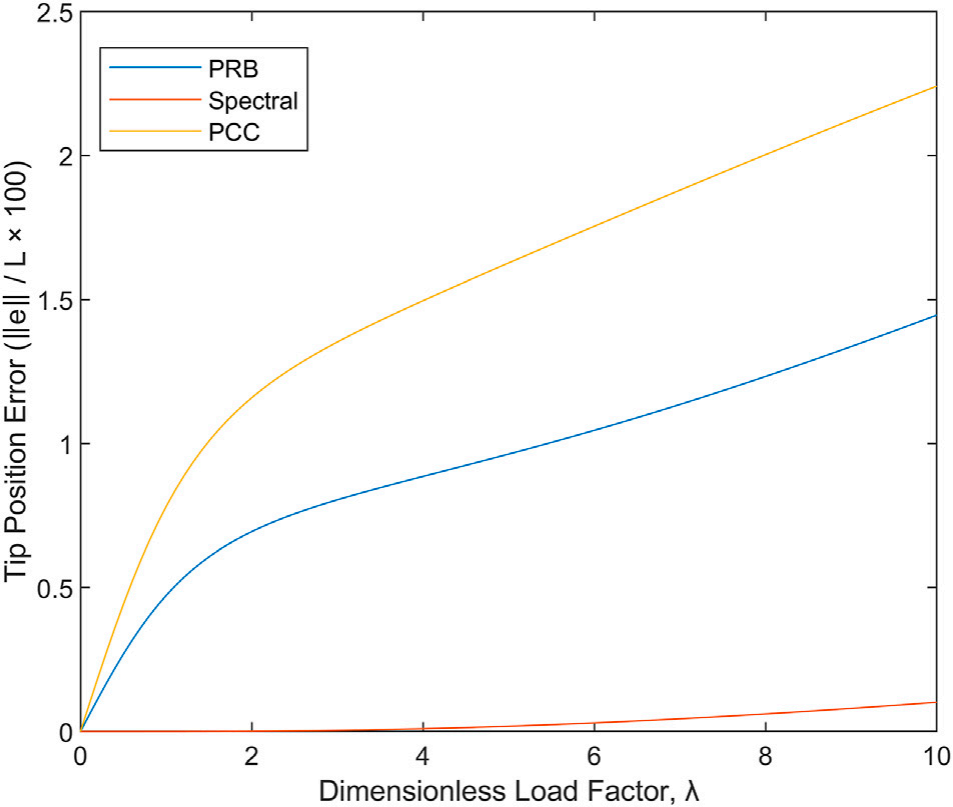
Error in reproducing the correct behavior under cantilevered loading conditions for three-DOF kinematic hypotheses of the PCC, PRB, and spectral types.

How well does each of the strategies perform when given 3-DOF to capture the deformation? The answer is depicted in Figure 6, showing tip position error in percent of robot length versus the dimensionless cantilevered load index λ . For cantilevered loads, a single spectral element which is quadratic in u is a far better choice than either a 3-element PCC model or a 3R PRB model. If nonzero shear forces are expected, the PCC model seems to have little in its favor; the Jacobian for the PRB model is simpler to calculate, meaning that the statics equations in Eq. 5 are easier to formulate, and the tip position is predicted more accurately, which also implies that the overall structural stiffness is more accurate for the PRB model than for the PCC model. The Jacobian for the spectral model, unlike the other two, cannot be obtained in an exact closed form.

The results imply that the typical piecewise constant-curvature assumption used in the development of geometrically nonlinear models for robots is a poor choice from the perspective of mechanics whenever a concentrated external load is present and is expected to produce internal shear forces which are transverse to v . In summary, if point loads are present on the robot, a linear interpolant of internal moment (equivalently curvature) is necessary to accurately capture the static equilibrium configurations for unrefined elements *even in the small deflection case*, and degrees of freedom are better spent on increasing the order of the interpolants than on increasing the number of elements.

#### Considerations for Non-Uniform Flexural Rigidity

In the case of non-uniform flexural rigidity, element refinement is more effective at reducing approximation error than increases in order. This conclusion is easily justified by the observation that if K(s) is a linear function, say for example 1+as , then in the simplest planar case with a constant internal moment one would be tasked to find another polynomial function κ(s) such that κ(s)K(s)=C for some constant C . But this is obviously impossible, because κ=C/K is a rational function, not a polynomial, and the Maclaurin series at s=0 has a finite region of convergence. In the example case, the expansion is $C\cdot \sum {\left(-1\right)}^{i}a^{i}s^{i}$ . The series does not converge unless |s|<|1/a| and as s approaches this upper bound, the number of terms in the series required to obtain convergence to a fixed tolerance increases without bound. Element refinement, on the other hand, has exactly the effect of reducing |s| , ensuring convergence. For this reason, single-element, spectral-type methods are not recommended as a first choice if non-uniform flexural rigidity is present.

#### Considerations for Uniformly Distributed Loads

Distributed loads may act on biomedical continuum robots. The most obvious of these loads is a gravitational force distributed along the length of the robot. Other common forces may include buoyancy forces, electric forces, magnetic forces, and aerodynamic and hydrodynamic forces. The simplest possible model of a distributed load is a uniform one that is applied normal to the body of a robot which is initially in a straight configuration. In this case, the solution to the linearized Euler-Bernoulli equation is in general a fourth-order polynomial in position. The shear force is a linear function of arc length and the internal moment (and hence curvature in the linear elastic case) is quadratic. If the shape is discretized at the level of angle, the discretization should be cubic to accommodate a uniform load.

#### Considerations for Elastic Environmental Contact

**FIGURE 7:**
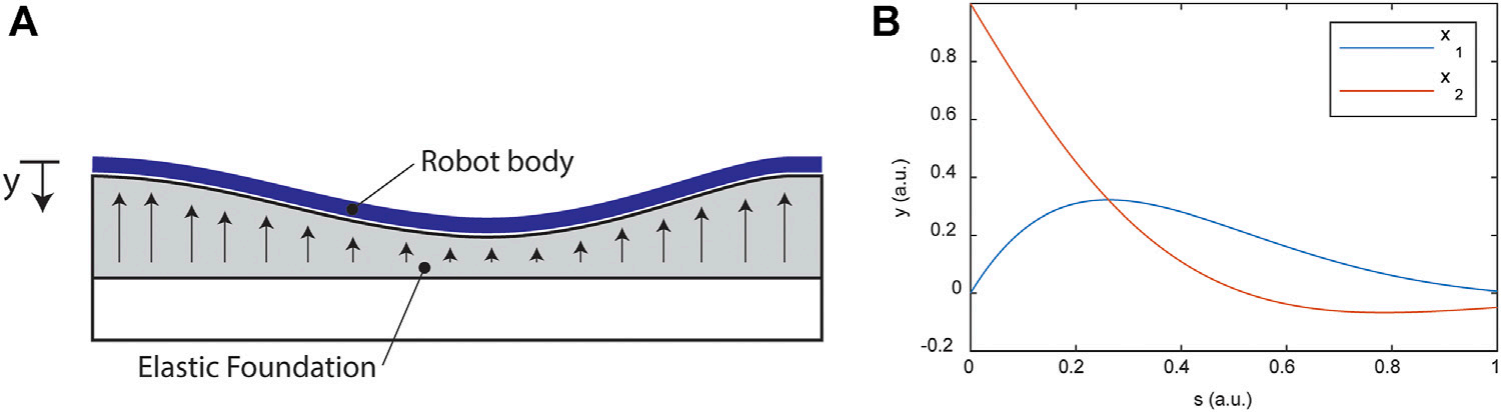
**(A)** Schematic diagram for the beam on an elastic foundation as a model for a continuum robot interacting with soft tissue. **(B)** Example with β=3 , showing the shape of the displacement that must be approximated.

For continuum robots in contact with soft bodies such as the soft tissues of the human anatomy, the contact might be well-described, at least in the region of contact, by a model like the linear elastic foundation model. For small deflections, the linearized Euler-Bernoulli model with a linear elastic foundation is modeled by the following differential equation (see Figure 7A).

$$\left(EIy{,}_{ss}\right){,}_{ss}=-ky.$$

The homogeneous solution to this equation has the following form.

$$y_{h}\left(s\right)=\exp\left(-\beta s\right)\left[C_{1}\sin\left(\beta s\right)+C_{2}\cos\left(\beta s\right)\right]+\exp\left(\beta s\right)\left[C_{3}\sin\left(\beta s\right)+C_{4}\cos\left(\beta s\right)\right].$$

The constant β depends only on the properties of the beam and the foundation.

$$\beta^{2}=\sqrt{\frac{k}{4EI}}.$$

To what degree of accuracy does a polynomial shape function (assuming the small-deflection case) approximate $y_{h}$ ?

To answer this question, one should find the best approximation of $y_{h}$ under a particular norm on $L^{2}\left[0,\ell \right]$ . Here we select the 2-norm and study the approximation error for 3^rd^, 4^th^, and 5^th^ order polynomials. Since β is related to the ratio of stiffnesses k and EI , and has dimension Length^−1^, we restrict the range of the dimensionless group ℓβ to (0.1, 10) . This range is consistent with the idea of *compliance matching* as a form of embodied intelligence in biomedical continuum robots. Note that as ℓβ→0 , the solution $x_{h}$ approaches a constant, which is easy to interpolate. As ℓβ→∞ , the elastic foundation is becoming infinitely stiff relative to the body of the robot, modeling a hard contact. In this case, the internal forces and moments and the resulting deformations become strongly localized, and a point load may be a more appropriate model for the contact than an elastic foundation.

The physical solutions to the equation decay away from the application of a point load. Therefore, we restrict the approximation problem to the consideration of the two functional forms that follow on a domain [0,1] for β∈(0.1,10) .

$$x_{1}=\exp\left(-\beta s\right)\sin\left(\beta s\right)\;\;x_{2}=\exp\left(-\beta s\right)\cos\left(\beta s\right).$$

See Figure 7B for examples with β=3 . Errors for polynomials $p_{1}$ and $p_{2}$ approximating $x_{1}$ and $x_{2}$ , respectively, are shown in Figure 4 as the maximum absolute errors.

$$e_{i}=\frac{\underset{s}{\max}\left|p_{i}\left(s\right)-x_{i}\left(s\right)\right|}{\underset{s}{\max}\left|x_{i}\left(s\right)\right|}$$

**FIGURE 8:**
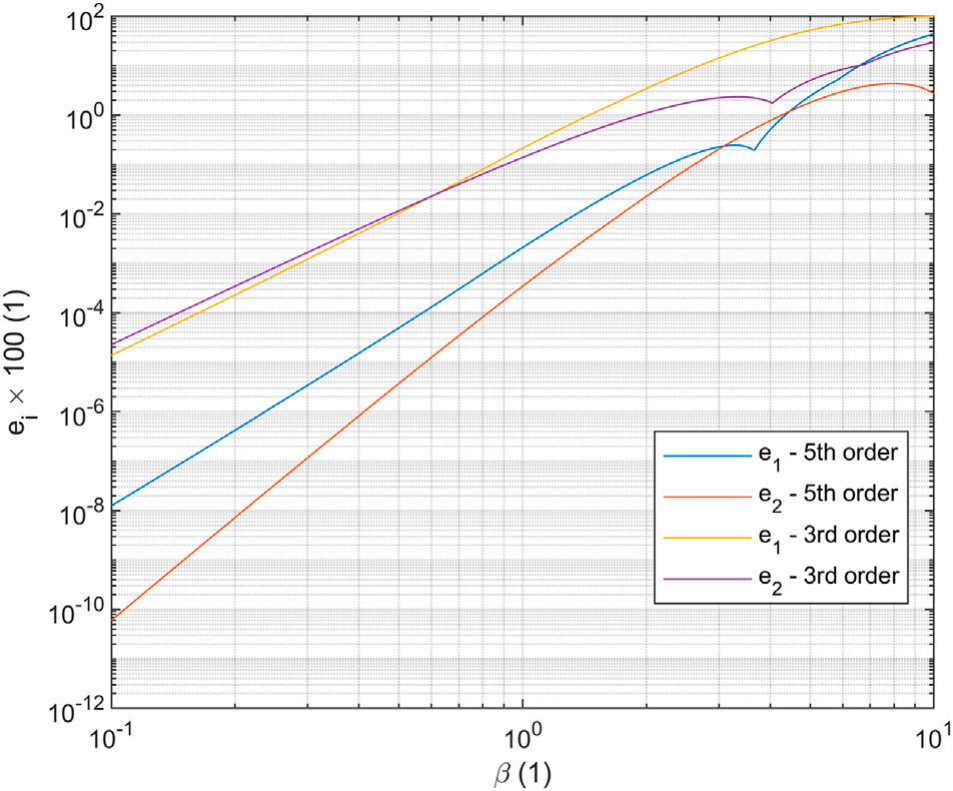
Approximation errors for best polynomial fits in the L^2^ norm to the solution for the linear beam on an elastic foundation problem. Higher-order polynomials permit greater elastic foundation stiffnesses.

For a single 5^th^ order polynomial in shape, the maximum absolute error in approximating either $x_{1}$ or $x_{2}$ remains below 1% if ℓβ<4.3 (Figure 8). As ℓβ increases beyond this value, the polynomial approximations to $x_{1}$ and $x_{2}$ begin to oscillate with increasing maximum error.

To put this in a practical perspective, a typical colonoscope has a linearized flexural rigidity of $EI\approx 0.02\;N\;m^{2}$ (Wehrmeyer et al., 1998). Soft tissues may have an elastic foundation stiffness of approximately 4 kN/m^2^ (Asadian et al., 2011). This results in β=15 and therefore a hypothesis which is 5^th^ order in position (3^rd^ order in strain variables) should not have elements longer than approximately 0.28 m. Note that for a hypothesis that is linear in s for the strain variables, the length requirement would drop to approximately 96 mm, and for PCC elements, the length would drop to only 38 mm. For a spatial robot model that is inextensible and un-shearable and is 1 m long, this would result in a PCC model with approximately 81 degrees of freedom (27 elements at 3 DOF/element), a linear strain variable model with approximately 66 degrees of freedom (11 elements at 6 DOF/element), or a cubic strain model with approximately 48 degrees of freedom (4 elements at 12 DOF/element). Therefore, if environmental contacts are soft and distributed over a long length, there is a strong incentive to develop models with higher-order strain variable hypotheses.

### Considerations for Numerical Methods

#### Solution Multiplicity

In general, the problem defined by Eq. 5 together with any constraints is a nonlinear algebraic problem, even if linear material models are used. This is either a consequence of the nonlinear geometry, which shows up in any finite-strain relationship between the strain variables and the position and orientation of the body, or a consequence of nonlinear material behavior, or both. In special cases, the problem may become linear; for example, if the actuators and generalized forces are related linearly, linear constitutive laws are used, and no external loads are present. For nonlinear static problems, the Newton-Raphson method and trust-region methods like the Levenberg-Marquardt method generally work well, but the modeler must be cautious of the possibility of solution multiplicity.

In other words, a function q=f(τ) does not always exist because there may be two or more values of q which satisfy the equilibrium conditions given τ . This solution multiplicity is accompanied by a singular tangent stiffness matrix for some value of q and possibly a tangent stiffness matrix with negative eigenvalues, as is the case for so-called “negative-stiffness mechanisms” (Platus, 1999). The coupling between kinematics and mechanics means that it is not always safe to assume the existence of a “forward kinematic mapping” which computes the C-space coordinates from the actuator variables and then the task-space variables from the C-space coordinates. Consider the case in which Eq. 5 is of the form $F\left(q,\tau ,Q_{nc}^{*}\right)=0$ where $Q_{nc}^{*}$ includes only those generalized forces which are not algebraically related with q and τ . Then a perturbation analysis yields the C-space Jacobians with respect to τ and $Q_{nc}^{*}$

δF=∂qF δq+∂τF δτ+∂QncF δQnc∗=0.

δq=−(∂qF)−1(∂τF δτ+∂QncF δQnc∗).

It is evidently at configurations with singular $\partial_{q}F$ where multiple solutions may arise. This is one reason that quasistatic “resolved-rate” or continuation-type methods may fail to converge; dynamic models do not suffer this problem since the accelerations are resolved.

#### Time Stepping

For time stepping, explicit ode integrators can become prohibitively computationally expensive. This is a consequence of the fact that unresolved vibrational modes (as defined for linear test problems) become unstable using explicit methods. Implicit integrators and those designed for solving stiff ODEs and DAEs, such as the trapezoidal method or the backwards difference formulae, are preferable. Energy-preserving integrators have the benefit that the damping behavior is caused entirely by the material model, ensuring repeatable dynamic behavior with different time steps.

### Current and Future Challenges in Modeling

#### Generalizability and Re-Usability

Despite the growing body of evidence that models built on the foundation of the Cosserat rod equations are an adequate description of many continuum robots, one challenge that still faces practitioners is a lack of standardized tools to build new model simulation codes. For rigid robots, a wide variety of domain-specific modeling languages are available and permit concise descriptions within an easy-to-use interface to build new models. One example of this is the Universal Robot Description Format and Gazebo simulator within the Robot Operating System, but there are many others presently available including Simulink/Simscape, Dymola, and other Modelica-language based toolsets such as OpenModelica (Brück et al., 2002; Fritzson et al., 2006; Miller and Wendlandt, 2011; Sucan and Kay, 2019). To enable the widespread re-use of validated modeling components, a library of reusable “model building blocks” for continuum robots should be designed. Some important capabilities of such a library would be the following:

• Coupling of curve-based models to rigid multibody models.
• Coupling of curve-based models and general finite element models.
• Incorporation of common actuator models.
• Incorporation of common constraints (length, concentricity, no-penetration, selective inextensibility/strong anisotropy, revolute and prismatic joints, etc.)
• User-selected switching between dynamic and quasi-static model generation.

For biomedical continuum robots in particular, models which couple to mechanical models of human anatomy are needed. Coupling of state-of-the-art models for continuum robots or their direct incorporation with real-time finite element codes using GPU acceleration is a promising approach (Allard et al., 2007; Duriez and Bieze, 2017).

#### Novel Kinematic Hypotheses

There is a great deal of freedom in element-based kinematic hypotheses which has yet to be explored. One interesting avenue is the use of a shared or constrained DOF between elements. The motivation for this idea is that for dynamic models, time stepping is sometimes restricted or difficult for “stiff” problems having many eigenvalues. The equations of motion for solid continua are wave equations, which means that if many elements are stacked end-to-end, acoustic waves (axial compression and tension) and twist waves (torsional waves) through the structure may be resolved by the model. For most robotics applications, these modes are likely to be irrelevant, and constraining the problem so that they do not exist in the model may improve computational performance. The elimination of twist waves in elastic rod models was previously considered by an energy minimization argument (Bergou et al., 2008).

Furthermore, adaptive kinematic hypotheses based on pre-defined, switchable degrees of freedom that permit local, automatic refinement of the model may allow greatly improved computational efficiency in problems involving a-priori unknown environmental interactions or constraints. This will permit, for example, a single high-order element to describe the deformation in free-space, while local refinement can take place where a catheter contacts a vessel wall, a robotic endoscopic system contacts the colon, or where multi-fingered hands contact an object to manipulate it.

#### Learning

Within the context of continuum and soft robotics, data-driven methods have begun to demonstrate strong utility. For example, Long Short Term Memory networks can capture hysteresis in pneumatically actuated catheters (Wu et al., 2021), and offline simulation of first-principles models can be used to learn reduced-order models using the snapshot-based proper orthogonal decomposition, resulting in new models suitable for real-time control and other applications requiring fast computation (Goury and Duriez, 2018; Katzschmann et al., 2019b). The continued development of learning methods enabling low-DoF representations will be an important future area of research.

There are also interesting opportunities for learning that amalgamate first-principles models with data-driven model “correctors,” or which use constrained learning techniques to identify models which are topologically like a curve-based model. One possibility is to use a low-DOF curve-based model capturing some of the behavior and to introduce a nonconservative generalized force $Q_{nc}$ which is learned from observed data to close the gap between simulation and reality. Learning-based methods which are constrained to obey fundamental principles are another emerging area of research, such as learning the Lagrangian or Hamiltonian function of systems directly from data (Ahmadi et al., 2018; Lutter et al., 2018).

#### Dynamic Model Validation

Although many dynamic models have been proposed, the validation of these models is currently lacking. There are many opportunities for rigorous evaluation and comparison of models with experimentally obtained data. The best and strongest form of model validation would be to instrument real robots with enough sensors to measure all the quantities appearing in Eq. 5 or the equivalent formulations for PRB and general continuum models, and to calculate the model residuals over conditions ranging over static, low-acceleration, and high-acceleration (e.g. sudden contact) regimes. This is clearly a challenging experimental task that may require state reconstruction and many sensors just to measure the configuration trajectory q(t) . Other options for validation may include comparison of standard test signal response characteristics (e.g. rise time, percent overshoot, settling time, steady-state error, and oscillation period) in response to both actuator inputs and environmental perturbations.

There are also many other interesting questions that can be asked and answered which are quantitative in a different sense, but which may be even more aligned with the spirit of soft and continuum robotics theory. For example, a model and simulated controller could be used to predict the success or failure of the navigation of a robotic catheter through tortuous vasculature parameterized by some measure of “tortuosity,” and then the classification error could be assessed via experiment matching the simulations.

## Conclusion

Continuum robots offer solutions to problems in biomedical applications which may not be solvable by traditional robotics technologies. With these new robots came the need for new models. A wide variety of physics-based and learning-based approaches to the modeling of continuum manipulators—both those made of hard materials and soft materials—are now available to the roboticist who needs them. This can lead to a dizzying array of choices for the uninitiated. This manuscript has reviewed the state-of-the-art approaches using a common language, discussed considerations which can guide the modeler when selecting which methods to use and some numerical difficulties to be aware of, and offered a view of the current and future challenges in the modeling of continuum robots. As modeling techniques continue to improve in terms of predictive power, as techniques begin to standardize, and as system identification techniques for soft and continuum robots mature, there is every reason to expect that the field will continue to expand, find new applications, and ultimately lead to transformative robotic solutions for human problems.

## Data Availability

The raw data supporting the conclusion of this article will be made available by the authors, without undue reservation.

## Footnotes

1 There are additional considerations for this placement in the case of dynamical models, discussed below.
